# Supplementary material for: Heterogeneity in lung macrophage control of Mycobacterium tuberculosis is modulated by T cells
Source: Nat Commun. 2024 Jul 8;15:5710. doi: 10.1038/s41467-024-48515-7 (PMC11231272; doi:10.1038/s41467-024-48515-7)
Supplement: Supplementary file 1 — Supplementary Information [file 41467_2024_48515_MOESM1_ESM.pdf]

Supplementary Figure 1

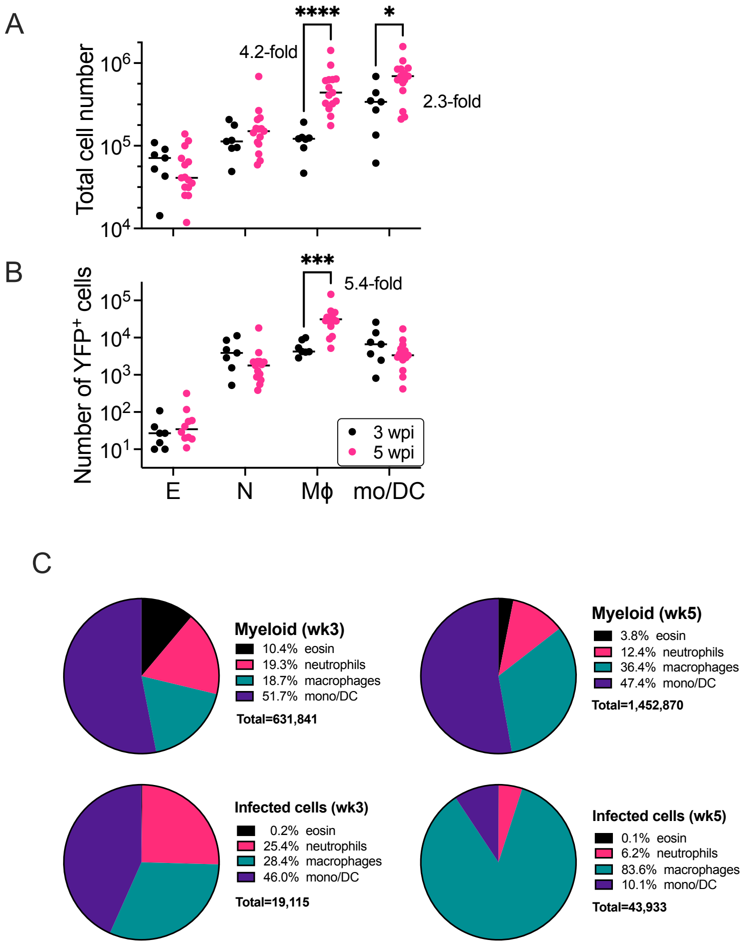

Supplementary Figure 1. Distribution of *M. tuberculosis* within myeloid cells. (A) Total numbers of each myeloid cell population at 3- and 5-weeks post low dose aerosol infection with Rv.YFP. (B) Total number of YFP$^+$ cells within each myeloid cell population. (C) Each cell population as a fraction of the total lung myeloid cells at three and five weeks post infection (top) and the number of infected cells within each myeloid population as a fraction of the total infected cells (bottom). These data represent two experiments with a total of n=7 mice (3 wpi) and three experiments with n=15 mice (5 wpi). Each point represents an individual subject and the line is the median. A two-way ANOVA with Šídák's multiple comparisons test was used to determine significance. *, p<0.05; ***, p<0.001; ****, p<0.0001. The numbers plotted in the pie charts are the medians. Numbers in A and B are the fold change between three and five weeks.

Supplementary Figure 2

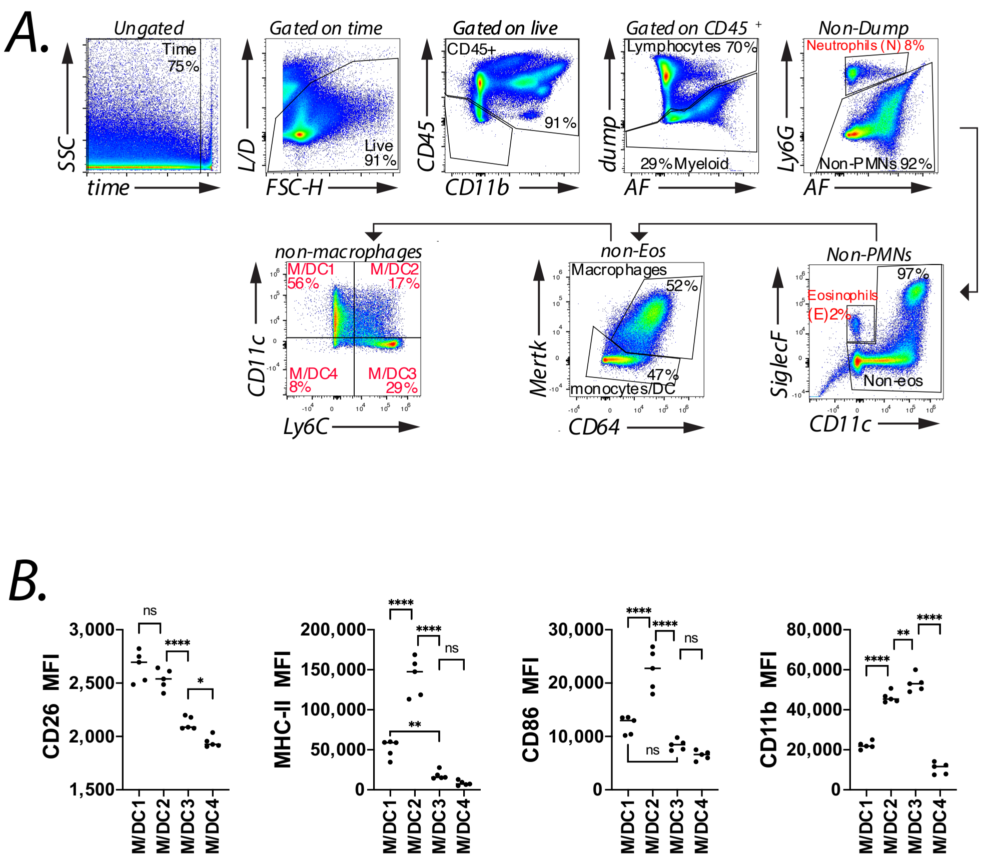

Supplementary Figure 2. Analysis of monocytes and DC. (A) Modified from Figure 2A. Gating strategy for identifying the different myeloid cell populations. In brief, a viability dye was used to exclude dead cells; CD45 and CD11b were used to exclude non-hematopoietic cells; CD3, CD19 and NK1.1 (vs. autofluorescence, AF) were used to exclude lymphocytes from the non-lymphoid hematopoietic cells. Ly6g and AF allowed identification of neutrophils; similarly, CD11c and SiglecF identified the eosinophils. Mertk and CD64 were used to separate macrophages from monocyte/DC populations. CD11c and Ly6c were used to subset the monocyte/DC populations into four populations labeled M/DC1 (Ly6c^{-}CD11c^{+}), M/DC2 (Ly6c^{+}CD11c^{+}), M/DC3 (Ly6c^{+}CD11c^{-}), M/DC4 (Ly6c^{-}CD11c^{-}), respectively. (B) The expression level of CD26, MHC-II, CD86, and CD11b by the four M/DC populations are expressed as MFI. Each point represents an individual subject (n=5). A one-way ANOVA with Šídák's multiple comparisons test was used to determine significance. *, p<0.05; **, p<0.01; ****, p<0.0001; ns, not significant. Bar, median.

Supplementary Figure 3

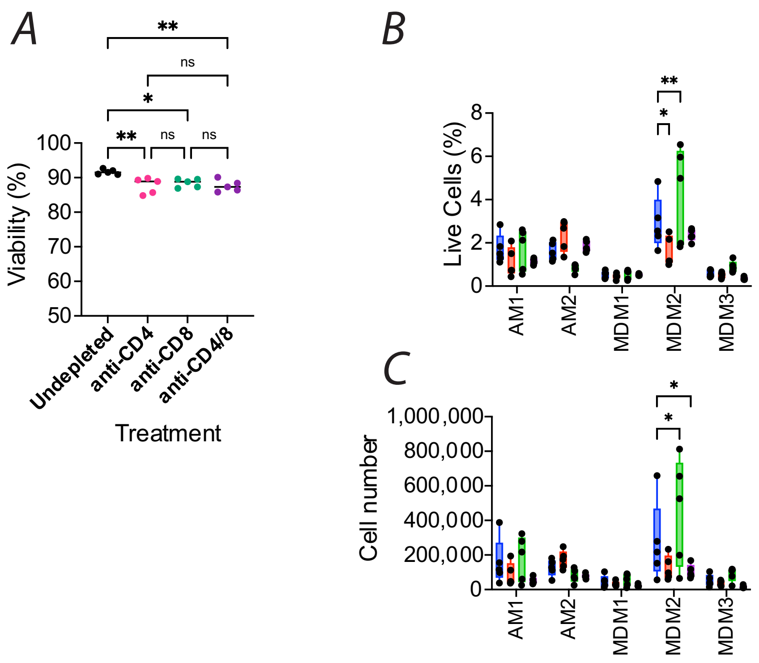

Supplementary Figure 3. Analysis of myeloid cells after T cell depletion. (A) The myeloid cell population was gated as described in Figure 2. Viability was assessed using Live/dead NIR (see methods). A one-way ANOVA with Tukey's multiple comparisons test was used to determine significance. *, p<0.05; **, p<0.01; ns, not significant. Bar, median. Representative data is shown for one experiment (n=5). (B) The frequency of different macrophage subsets among total live cells and their absolute cell number after administration of antibodies to CD4, CD8, or both. (B, C) Each point represents an individual subject (n=5). A one-way ANOVA with Šídák's multiple comparisons test was used to determine significance. *, p<0.05; **, p<0.01; ****. Bar, median.

Supplementary Figure 4

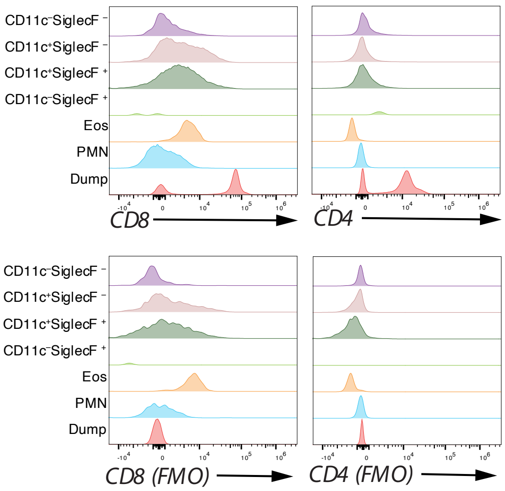

Supplementary Figure 4. Expression of CD4 and CD8 on the different myeloid cell subsets identified in the study. CD4 and CD8 expression were examined on various cell subsets in the lung at 5 weeks post Rv.YFP infection. Dump population includes B cells, T cells and NK cells. Top panels show the relative expression of CD8 and CD4 in each population, with the corresponding FMOs below.

Supplemental Table 1. List of antibodies used in the study.

Fluor	**Marker**	**Clone**	**Dilution**
BV421	CD19	6D5	1/200
	CD90.2	30-H12	1/600
	NK1.1	PK136	1/100
PacBlue	CD11c	N418	1/150
BV605	Ly6G	1A8	1/100
BV650	CD26	H194-112	1/50
BV786	SiglecF	E50-2440	1/200
PerCP Cy5.5	Ly6C	HK1.4	1/133
PE	Mertk	2B10C42	1/50
AF594	CD11b	M1/70	1/250
PE-Cy5	CD86	GL-1	1/50
PE-Cy7	CD64	X54-5/7.1	1/250
SparkNIR 685	CD45.2	104	1/200
AF700	MHCII	M5/114.15.2	1/50
APC	iNOS	CXNFT	1/200
NIR	LiveDead		0
Fc			1/200
