# Supplementary material for: Heterogeneity in lung macrophage control of Mycobacterium tuberculosis is modulated by T cells
Source: Nat Commun. 2024 Jul 8;15:5710. doi: 10.1038/s41467-024-48515-7 (PMC11231272; doi:10.1038/s41467-024-48515-7)
Supplement: Supplementary file 3 — Reporting Summary [file 41467_2024_48515_MOESM3_ESM.pdf]

nature portfolio

Corresponding author(s): Sam Behar, Rocky Lai

Last updated by author(s): Feb 27, 2024

Reporting Summary

Nature Portfolio wishes to improve the reproducibility of the work that we publish. This form provides structure for consistency and transparency in reporting. For further information on Nature Portfolio policies, see our Editorial Policies and the Editorial Policy Checklist.

Statistics

For all statistical analyses, confirm that the following items are present in the figure legend, table legend, main text, or Methods section.

n/a	Confirmed	
☐	☒	The exact sample size (n) for each experimental group/condition, given as a discrete number and unit of measurement
☐	☒	A statement on whether measurements were taken from distinct samples or whether the same sample was measured repeatedly
☐	☒	The statistical test(s) used AND whether they are one- or two-sided<br>*Only common tests should be described solely by name; describe more complex techniques in the Methods section.*
☐	☒	A description of all covariates tested
☐	☒	A description of any assumptions or corrections, such as tests of normality and adjustment for multiple comparisons
☐	☒	A full description of the statistical parameters including central tendency (e.g. means) or other basic estimates (e.g. regression coefficient) AND variation (e.g. standard deviation) or associated estimates of uncertainty (e.g. confidence intervals)
☐	☒	For null hypothesis testing, the test statistic (e.g. F, t, r) with confidence intervals, effect sizes, degrees of freedom and P value noted<br>*Give P values as exact values whenever suitable.*
☒	☐	For Bayesian analysis, information on the choice of priors and Markov chain Monte Carlo settings
☒	☐	For hierarchical and complex designs, identification of the appropriate level for tests and full reporting of outcomes
☒	☐	Estimates of effect sizes (e.g. Cohen's d, Pearson's r), indicating how they were calculated

Our web collection on statistics for biologists contains articles on many of the points above.

Software and code

Policy information about availability of computer code

Data collection	Spectroflo Version 3.1 was used to collect flow data on Cytek Aurora.<br><br>Sony MA900 Cell Sorter Software v3.1.0 was used to sort AM and MDM2
Data analysis	FlowJo v10.10 was used to analyze flow data. Graphs were generated using Graphpad Prims v10.

For manuscripts utilizing custom algorithms or software that are central to the research but not yet described in published literature, software must be made available to editors and reviewers. We strongly encourage code deposition in a community repository (e.g. GitHub). See the Nature Portfolio guidelines for submitting code & software for further information.

Data

Policy information about availability of data

All manuscripts must include a data availability statement. This statement should provide the following information, where applicable:

- Accession codes, unique identifiers, or web links for publicly available datasets
- A description of any restrictions on data availability
- For clinical datasets or third party data, please ensure that the statement adheres to our policy

All data supporting the findings of this study are available within the paper and its supplemental information. Further information and requests for resources and reagents should be directed to and will be fulfilled by the Lead Contact, Samuel Behar (Samuel.Behar@umassmed.edu).

Research involving human participants, their data, or biological material

Policy information about studies with human participants or human data. See also policy information about sex, gender (identity/presentation), and sexual orientation and race, ethnicity and racism.

Reporting on sex and gender	n/a
Reporting on race, ethnicity, or other socially relevant groupings	n/a
Population characteristics	n/a
Recruitment	n/a
Ethics oversight	n/a

Note that full information on the approval of the study protocol must also be provided in the manuscript.

Field-specific reporting

Please select the one below that is the best fit for your research. If you are not sure, read the appropriate sections before making your selection.

[x] Life sciences [ ] Behavioural & social sciences [ ] Ecological, evolutionary & environmental sciences

For a reference copy of the document with all sections, see nature.com/documents/nr-reporting-summary-flat.pdf

Life sciences study design

All studies must disclose on these points even when the disclosure is negative.

Sample size	In vivo experiments: no statistical method was used to determine sample size. The number of mice used was increased as the experiments progressed to improve statistical power.
Data exclusions	No data was excluded for this study.
Replication	All data presented in the data was replicated at least once
Randomization	Experiments samples were compared between treatment groups and controls and therefore was not possible to randomize.
Blinding	Experiments were not blinded as all experimental groups were compared to control samples.

Reporting for specific materials, systems and methods

We require information from authors about some types of materials, experimental systems and methods used in many studies. Here, indicate whether each material, system or method listed is relevant to your study. If you are not sure if a list item applies to your research, read the appropriate section before selecting a response.

Materials & experimental systems

n/a	Involved in the study	
[ ]	[x]	Antibodies
[x]	[ ]	Eukaryotic cell lines
[x]	[ ]	Palaeontology and archaeology
[ ]	[x]	Animals and other organisms
[x]	[ ]	Clinical data
[x]	[ ]	Dual use research of concern
[x]	[ ]	Plants

Methods

n/a	Involved in the study	
[x]	[ ]	ChIP-seq
[ ]	[x]	Flow cytometry
[x]	[ ]	MRI-based neuroimaging

Antibodies

Antibodies used

Reagent or resource Source Identifier
Antibodies
Live Dead NIR Invitrogen L10119A
CD45 Spark NIR (clone 104) Biolegend 109864
CD11b Alexa Fluor 594 (clone M1/70) Biolegend 101254

	CD90.2 BV421 (clone 30H12) Biolegend 105341<br>CD19 BV421 (clone 6D5) Biolegend 115549<br>NK1.1 BV421 (clone PK136) Biolegend 108731<br>CD26 BV650 (clone H194-112) BD 740474<br>CD86 PE-Cy5 (clone GL-1) Biolegend 105016<br>Ly6G BV605 (clone 1A8) Biolegend 127639<br>SiglecF BV786 (clone E50-2440) BD 740956<br>CD11c Pacific Blue (clone N418) Biolegend 117322<br>Mertk PE (clone 2B10C42) Biolegend 151506<br>CD64 PE-Cy7 (clone x54-5/7.1) Biolegend 139314<br>Ly6c PerCP-cy5.5 (clone HK1.4) Biolegend 128012<br>anti-CD4 (clone GK1.5) Bioxcell BE0003-1<br>anti-CD8 (clone 2.43) Bioxcell BE0061
Validation	All the of the antibodies used in this study were obtained commercially and their validation was done by the individual companies. In addition, we validated the antibodies based on know positive staining patterns. The anti-CD4 and anti-CD8 mAb that were used for depleting T cell subsets were further validated in our hands based on their ability to deplete CD4 and CD8 lymphocytes, respectively, as determined by flow cytometry as described in the manuscript.<br><br>Companies that sells the antibodies we used provide quality certificate that certifies these antibodies have been manufactured and tested in accordance with their specifications. The catalog numbers of the antibodies are provided so that each antibodies can be found on the website of each company.<br>Quality Control:<br>BioLegend: https://www.biolegend.com/en-gb/quality<br>Thermo Fisher Scientific: https://www.thermofisher.com/us/en/home/life-science/antibodies/invitrogen-antibody-validation<br>BD Biosciences: https://www.bdbiosciences.com/en-us/products/reagents/flow-cytometry-reagents/research-reagents/quality-and-reproducibility<br>Bioxcell: https://bioxcell.com/what-we-do/

Animals and other research organisms

Policy information about studies involving animals; ARRIVE guidelines recommended for reporting animal research, and Sex and Gender in Research

Laboratory animals	Mice. 6-8 week old mice were purchased from Jackson Laboratories (bar Harbor, ME). RAG1-/- mice were bred in housed under specific-pathogen free (SPF) conditions or higher.<br><br>Standard animal room lighting for most species is set to have light from 7 AM to 7 PM daily, and dark from 7 PM to 7 AM. Adjustments are made for daylight savings time. Room temperature, humidity and ventilation are based on the recommendations from the Guide for the Care and Use of Laboratory Animals of the National Institutes of Health and the Office of Laboratory Animal Welfare.
Wild animals	No wild animals were used in the study
Reporting on sex	Only female mice were used in this study as sex was not a consideration in this study.
Field-collected samples	No field collected samples were used in the study.
Ethics oversight	Studies studies involving animals were conducted following relevant guidelines and regulations, and the studies were approved by the Institutional Animal Care and Use Committee at the University of Massachusetts Medical School (Animal Welfare A3306-01), using the recommendations from the Guide for the Care and Use of Laboratory Animals of the National Institutes of Health and the Office of Laboratory Animal Welfare.

Note that full information on the approval of the study protocol must also be provided in the manuscript.

Plants

Seed stocks	n/a
Novel plant genotypes	n/a
Authentication	n/a

Flow Cytometry

Plots

Confirm that:

- [x] The axis labels state the marker and fluorochrome used (e.g. CD4-FITC).
- [x] The axis scales are clearly visible. Include numbers along axes only for bottom left plot of group (a 'group' is an analysis of identical markers).
- [x] All plots are contour plots with outliers or pseudocolor plots.
- [x] A numerical value for number of cells or percentage (with statistics) is provided.

Methodology

Sample preparation	Lung cell preparation. To isolate total lung leukocytes, lungs were perfused by slowly injecting PBS into right ventricle immediately after mice were killed. The lungs were minced with a gentleMACS dissociator (Miltenyi) and digested (30 min, 37°C) in 250 U/ml collagenase and 60 U/ml DNase (both from Sigma-Aldrich). Lung cell suspensions were passed through a 70-µm and 40-µm strainers sequentially to remove cell clumps. Lung cells were resuspended in autoMACS running buffer (Miltenyi) that contains BSA, EDTA, and 0.09% sodium azide for subsequent staining.<br><br>Flow cytometry analysis. Cells were first stained with Live/Dead Fixable NIR Dead cell Stain Kit (ThermoFisher) for 10 minutes at room temperature (RT), after which cells were stained with 5 ug/ml of anti-mouse CD16/32 mAb (BioXcell) in autoMACS running buffer (Miltenyi) for 10 minutes at 4°C. Next, the cells were then stained with a surface antibody cocktail for 20 minutes at 4°C. The antibodies included a dump channel was used to exclude T cells, B cells, and NK cells for efficient myeloid cell analysis and included anti-Thy1.2 (clone 30H12), anti-CD19 (clone 6D5), and anti-NK1.1 (clone PK136) with PE-Cy7. Macrophages were subsequently defined based on a combination of CD11c (clone N418), SiglecF (clone E50-2440), Mertk (clone 2B10C42), CD64 (clone X54-5/7.1), Ly6C (clone HK1.4). To inactivate the bacteria, samples were fixed with 1% paraformaldehyde/PBS for 1 hour at room temperature and then washed with MACS buffer. Samples were run on either a 4 or 5 laser Cytek Aurora. Erdman (non-fluorescent) infected mice used for unstained control, single stains and YFP-FMO. Autofluorescence (AF) was treated as a fluorescent parameter during unmixing either automatically through the SpectralFlo software or by manually deriving an autofluorescence fingerprint (assigned to BV510). After unmixing FMOs were used to define positive and negative populations for each parameter. Flow data were analyzed using FlowJo v10.7.1.
Instrument	Cytek Aurora 5 laser configuration
Software	SpectroFlo software was used for data collection. Data analysis was performed using FlowJo v10.7.1
Cell population abundance	AM and IM populations were identified as described in Figure 2A and sorted using an MA900 Sony Cell sorter. Cells were sorted under normal mode which yields approximately 95% purify of identified population.
Gating strategy	Gating strategy can be found in both Figure 2 as well as supplemental Figure 2.

- [x] Tick this box to confirm that a figure exemplifying the gating strategy is provided in the Supplementary Information.
